# Radical Observations about Teeth

**Published:** 1873-11

**Authors:** 


### RADICAL OBSERVATIONS ABOUT TEETH.

TO THE EDITOR OF THE DENTAL REGISTER:

I wonder if a few radical observations upon people's teeth and mouths would be in order, from an unscientific and irregular correspondent, though an occasional reader of the RE[illegible]-

ISTER? If so, I shall take the liberty to say what I have in my own way. Pardon the egotism, but I plume myself upon not only being a good observer, but upon having enormous opportunities for observation; and I have also a *penchant* for looking close at people—into their faces, eyes and mouths—as far as I can without offensive obtrusiveness.

I have to observe then, by your leave, that there is an amazing amount of disgusting carelessness about the mouth and teeth. I see men and women every day ordinarily endowed with intelligence, and who have acquired means enough not only to live comfortably, but to entertain, perfectly ignorant of or careless about their teeth. They have the temerity to look you in the face and talk to you within nosing distance, perfectly oblivious of the fact that you have an olfactory nerve. Smiling belongs as a general thing to women and politicians, that is after the period of childhood is passed, but it is really pitiful to observe the futile efforts of sunny-natured women to conceal the hideousness of neglected teeth in the struggle of a smile. In full toilette the fan serves a useful purpose, but, generally speaking, the hand, a book, or a turning away of the head during the cachinatory impulse, are mainly resorted to, and the sweetness of the smile is positively destroyed by the advanced progress of decay. I cannot understand how women with neglected teeth can pass through the ecstatic period of "love's young dream," win a husband and become the mother of children without creating insuperable disgust to the most intimate relation that nature claims, religion sanctions, and society binds and prospers by. I find too, in my mental notes, a singular indifference to appearance on the part of public speakers, such as lawyers and preachers of the gospel. In the former it is more excusable or rather less reprehensible, for their appearance is only before the morbid and low, harried wretches who hang about courts of justice, "Heaven save the mark," like bats in old ruins or in caves of the earth; but there is no excuse whatever for the latter class, to whom the people not only look for precept, but for example in whatsoever things are lovely. I know fellows in the pulpit with whom physical contact is loathsome. They almost kill at rifle shot distance. It is wonderful that such easy lessons as Shakspeare taught over three hundred years ago, by the lips of a rude mechanic, are so easily forgotten or overlooked. He makes glorious Nick Bottom adjure the hard-handed men of Athens thus: "And most dear actors, eat no onions or garlic, for we are to utter sweet breath." How can the oracle of God utter sweet breath with a mouth full of bad teeth, when it was essentially requisite in a parcel of clowns about to enact a play before an earthly prince?

The grandest character in the New Testament history, writing to the Corinthians said: "What! know ye not that your body is the temple of the Holy Ghost, which is in you?" I do not hesitate to affirm that Paul used his nail and tooth brushes regularly, and that he was a very model of personal neatness. He was a "marvellous proper man." But to return to my observations. Actors and lyric artists generally are careful of their teeth; indeed, so are that large class known as show people with whom personal appearance is almost everything.

The thoughtful and observant man of the world will recognize at once the important part that personal neatness plays in the most deplorable phase of social life, without any further reference to the subject than to remind the reader of the fact.

I shall never forget a serio-comic poem I heard many years ago, each stanza of which commenced with "She never smiled," and went on in the most dolorous strain about the gradual fading out of every hope until at last the face became fixed and settled into an expression of sadness as unchangeable as the visage of a statue, because, as the last line revealed, "her front teeth were decayed."

It is not my business here to persuade people to go to a dentist, even if they have good teeth, so as to keep them good, for no one is excusable now-a-days for ignorance on that point; I merely set out to say what I have observed about bad teeth, and I am quite sure I am not alone in that line of observation, for the mouth is one of the first features that fixes the attention of man or woman after the purpose and intent of the *vis a vis* are read in the eyes. E. B.

